# Co_3_O_4_/carbon composite nanofibrous membrane enabled high-efficiency electromagnetic wave absorption

**DOI:** 10.1038/s41598-018-30871-2

**Published:** 2018-08-17

**Authors:** Ibrahim Abdalla, Jiali Shen, Jianyong Yu, Zhaoling Li, Bin Ding

**Affiliations:** 1 State Key Laboratory for Modification of Chemical Fibers and Polymer Materials, College of Materials Science and Engineering, Donghua University, Shanghai, 201620 China; 2 Key Laboratory of Textile Science and Technology, Ministry of Education, College of Textiles, Donghua University, Shanghai, 201620 China; 3 Innovation Center for Textile Science and Technology, Donghua University, Shanghai, 200051 China

## Abstract

Electromagnetic (EM) wave absorbing materials have been fabricated from diverse materials such as conductive polymers, carbon based nanostructures and magnetic metal oxides. Nevertheless, it has remained a great challenge to develop lightweight and high-efficiency EM wave absorbing materials with a broad frequency range. Herein, we report a scalable strategy to create Co_3_O_4_/carbon composite nanofibrous membrane by electrospinning technique followed by stabilization and carbonization processes. An optimal reflection loss (R_L_) value of 36.27 dB is reached at 13.76 GHz for a layer of 2 mm thickness. RL exceeding −20 dB can be realized in any interval within the 4.5–14.4 GHz range by selecting a proper thickness of the absorbing layer between 1 and 5 mm. The Co_3_O_4_/carbon composite nanofibrous membrane could be well served as promising and attractive candidates for lightweight and enhanced EM wave absorbing materials. This presented research provides an innovative and effective approach to design the novel EM wave absorbing material in a broad frequency range for practical applications.

## Introduction

In recent times, with the rapid development of telecommunication and aerospace technology, electromagnetic (EM) wave absorption materials have gained great attention due to concerns about the negative effect of EM interference on the human body and military fields^1–3^. The increasing requirements of electronic devices and advancement of military stealth technology result in large demand of microwave absorbing materials^4^. EM wave absorption materials are capable of efficiently absorbing the EM waves and then convert them into thermal energy or dissipate the EM waves by interference^5^. These functional products are generally classified into dielectric and magnetic materials^6,7^. Development of materials with high-efficiency microwave attenuation performance can be beneficial to prevent the harm from EM waves released from electronic devices in both civil and military fields^8^.

As a significant ferromagnetic material, cobalt possesses high saturation magnetization, making it a better broadband microwave absorber^9^. Cobalt oxide is a semiconductor material exhibiting greater dielectric properties and has been widely used in electronic and bio-sensor areas^10^. Furthermore, cobalt oxide can be serving as an effective EM wave absorption material. Among transition metal oxides, cobalt oxide has the advantages of high electrochemical performance and environmental friendliness^11^. Meanwhile, carbon based materials are commonly used as lightweight EM wave absorption materials owing to their excellent features such as improved conductivity and favorable thermal stability^12,13^. Developing a composite absorber by loading the cobalt oxide into the porous carbon nanofibers will be a superior solution to ensure the fabrication of lightweight and high-efficiency EM wave absorbing materials.

According to the energy conversion principle, the absorbing performance of EM wave materials is greatly attributed to the relative complex permittivity (${\varepsilon }_{r}={\rm{\varepsilon }}^{\prime} -j{\rm{\varepsilon }}^{\prime\prime}$) and permeability (${\mu }_{r}=\mu ^{\prime} -j\mu ^{\prime\prime}$)^14^. A proper matching between the complex permittivity and permeability determines both of the reflection loss (R_L_) and attenuation characteristics of EM absorber^15,16^. The EM wave absorption capability depends largely on the nature, shape, and size of an absorber^5^. Qin^17^ reported that better EM wave absorbers were supposed to have lower reflection loss and a broad frequency range as well as being lightweight, thin and cost-effective. Considerable efforts have been devoted to exploring or examining the effect of cobalt oxide nanoparticles on the EM wave absorption properties. Wang^18^ prepared the CoNi/C nanocapsules absorber by a modified arc-discharge method, and a RL value of −35 dB was reached at a frequency range of 5–17 GHz. Han^19^ fabricated the FeCo/C composite by a modified arc-discharge technique, and a R_L_ value of −29 dB can be reached at a frequency range of 2–6 GHz. However, for the mentioned previous work, a wide adoption of this arc-discharge technique may be shadowed by unfavorable limitations such as sophisticated and expensive equipment, complex and time-intensive procedures as well as high opening cost^20^. Besides, the resultant composites exhibited relatively limited EM wave absorbing properties since the obtained materials had a lower R_L_ of the incidence wave. Consequently, it is highly meaningful and desirable to develop new approaches for EM wave absorption nanofibrous composite materials with low cost and improved performance.

In this work, we present a novel nanofibrous absorber composed of flexible carbon nanofibers and magnetic cobalt oxide nanoparticles by combined techniques of electrospinning, stabilization and carbonization. The as-synthesized composite exhibited enhanced EM wave absorption performance in a broad frequency range with smaller absorber layer thickness compared with previously reported magnetic composites. Given a collection of compelling merits of being flexible, easy fabrication, environmentally friendly and cost-effective, the fabricated materials not only represent a new and efficient pathway for high performance microwave absorption, but also a substantial advancement in the development of magnetic devices and could offer new design options for electromagnetic shielding system.

## Results and Discussion

**Figure 1:**
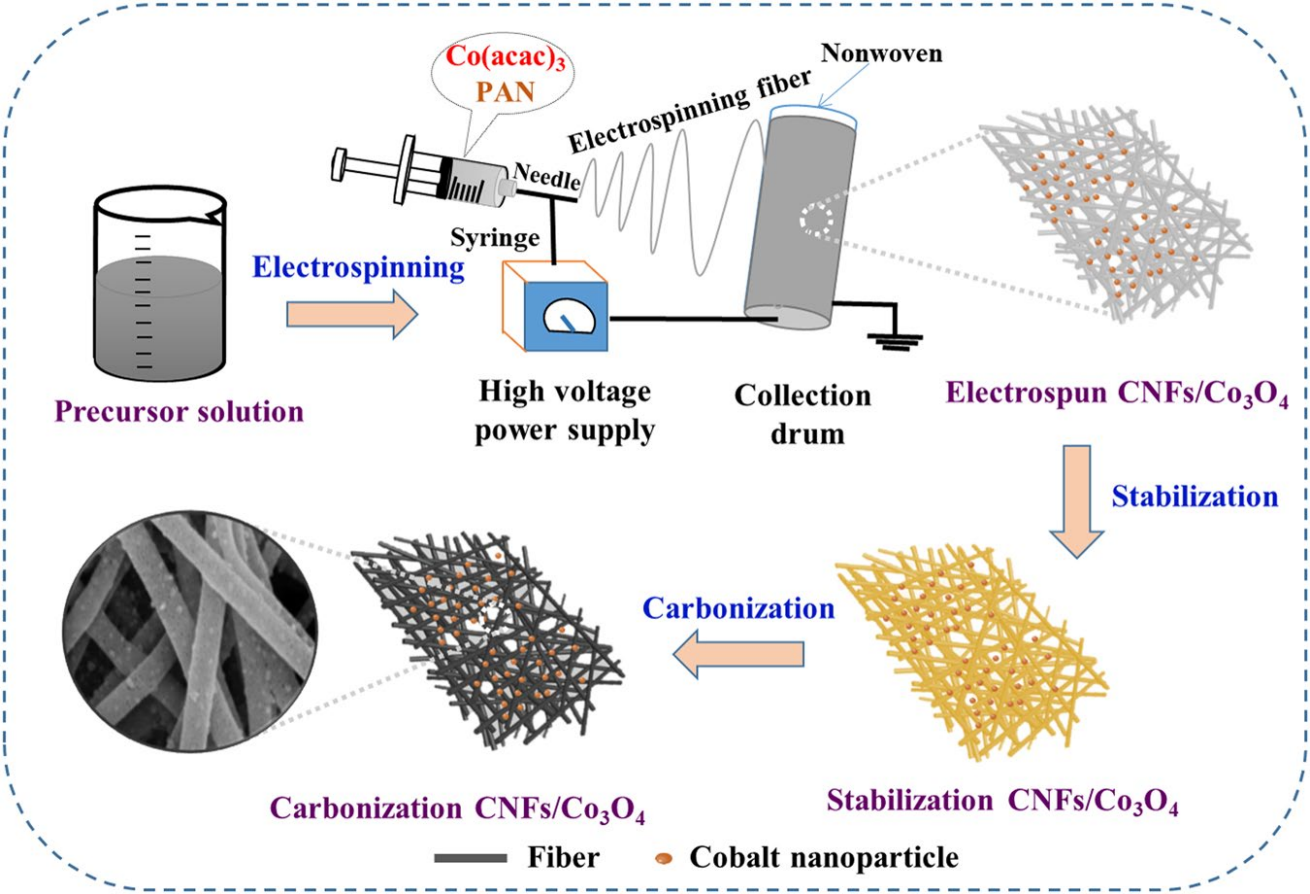
Schematic diagram showing the synthetic process of CNFs/Co_3_O_4_ composite samples.

The CNFs/Co_3_O_4_ composite samples were fabricated through changing the proportional amounts of cobalt acetylacetonate (AAc) into solutions. The PAN polymer nanofibers were annealed at 800 °C to improve the EM wave absorption of the final composite membrane. A schematic diagram of the synthetic process is depicted in Fig. 1 The precursor solution of PAN polymer with different concentrations of cobalt acetylacetonate was first prepared and the PAN/AAc nanofiber membrane was then formed by electrospinning technique. Afterwards, the obtained membrane was stabilized in an oven with external tension. The treated membrane was subsequently annealed in a tube furnace under N_2_ flow to complete the carbonization process and the black magnetic CNFs/Co_3_O_4_ composite membrane was finally obtained. The produced sample was denoted as CAc-K (c refers to AAc content; K refers to carbonized temperature) and pure carbon nanofibrous was known as CNFs-K. For comparison, the four samples of CA2, CA3, CA4 and CNFs were fabricated by using 1 g, 1.5 g, 2 g and 0 g of AAc respectively in the precursor solution. A detailed fabrication process of the resultant nanofibrous membrane is presented in the experimental section.

**Figure 2:**
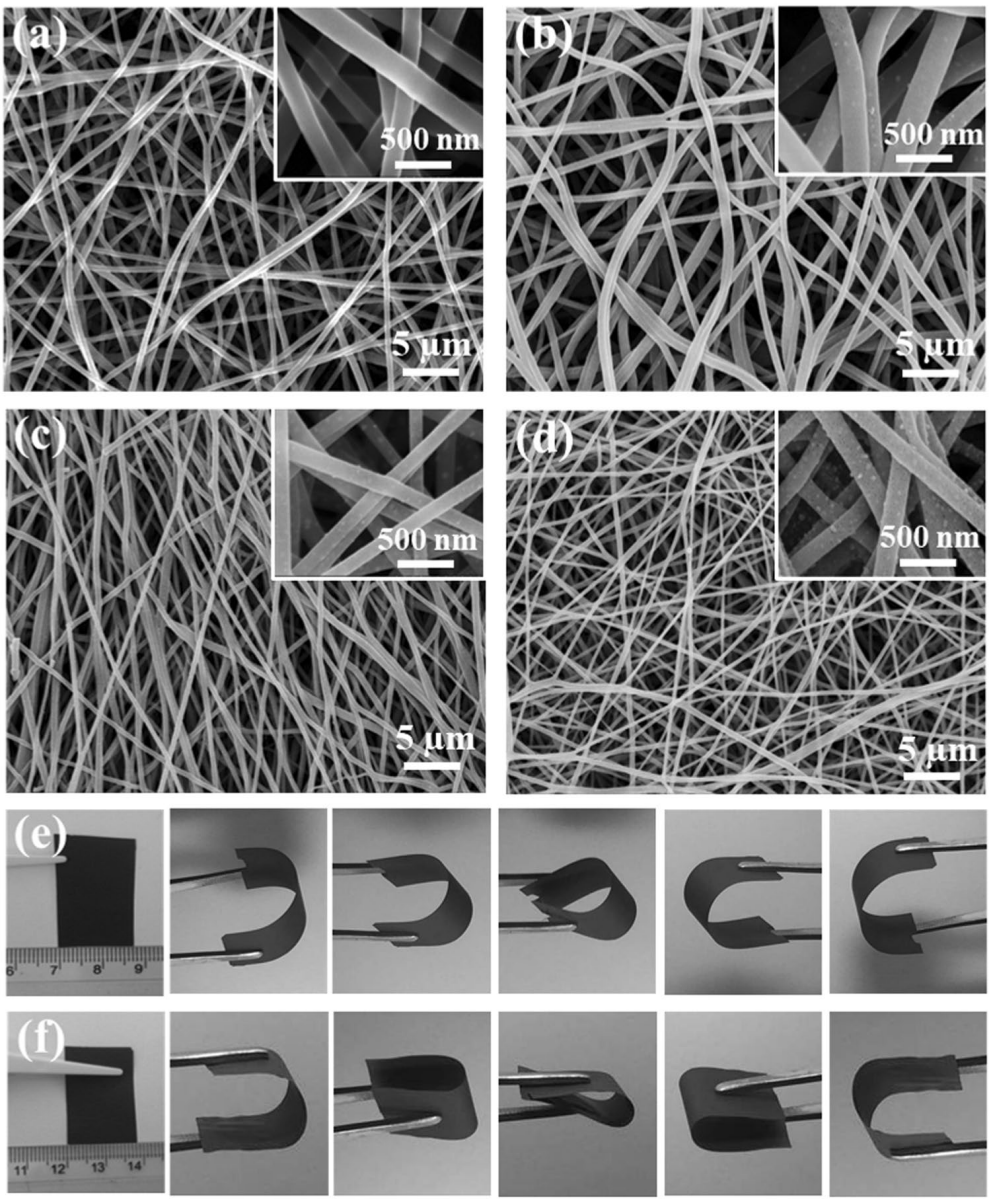
FE-SEM images of (**a**) CNFs-800, (**b**) CA2-800, (**c**) CA3-800, (**d**) CA4-800 and the digital images presenting the flexibility of (**e**) CA3-800 and (**f**) CA4-800 samples.

The morphologies of the cobalt oxide/carbon nanofiber samples were confirmed by Field Emission Scanning Electron Microscopy (FE-SEM) investigation. Figure 2 demonstrates FE-SEM images of the as-fabricated CNFs-800, CA2-800, CA3-800 and CA4-800, which indicated that the resultant nanofibers were randomly oriented and Co_3_O_4_ nanoparticles were uniformly distributed on the surface of the carbon nanofibers. The fiber diameters of CA2-800, CA3-800 and CA4-800 were 0.18 µm, 0.15 µm, and 0.11 µm, respectively. It is clearly showed that with an increase of AAc salt concentration, the diameter of the CNFs/Co_3_O_4_ composite membrane decreased accordingly (Fig. S1). The high-magnification SEM images seen in Fig. 2(b–d) evidently display that the Co_3_O_4_ nanoparticles are not only distributed uniformly on the surface of carbon nanofibers but also are regularly penetrated within the carbon nanofiber layers, benefiting from the doping of nanoparticles in the carbon matrix. The digital images indicating the flexibility of the CA3-800 and CA4-800 composite samples are presented in Fig. 2(e,f). Therefore, the digital images of (a) the CNFs-800 and (b) (CA2-800) are displayed in Fig. S2. As seen in the images, pure sample (CNFs-800) is more brittle while the lower concentration of nanoparticle sample (CA2-800) exhibits a lower flexibility compared with CA3-800 and CA4-800 composite samples. The high flexibility comes from the effect of high concentration of nanoparticles in the composite sample. (Movie S1) also presents the good flexibility of CA4-800 composite sample. Carbon nanofibers are significantly structural and useful materials owing to its excellent mechanical and electrical properties and thus widely used as reinforcements for better nanocomposites with enhanced performance^21,22^.

**Figure 3:**
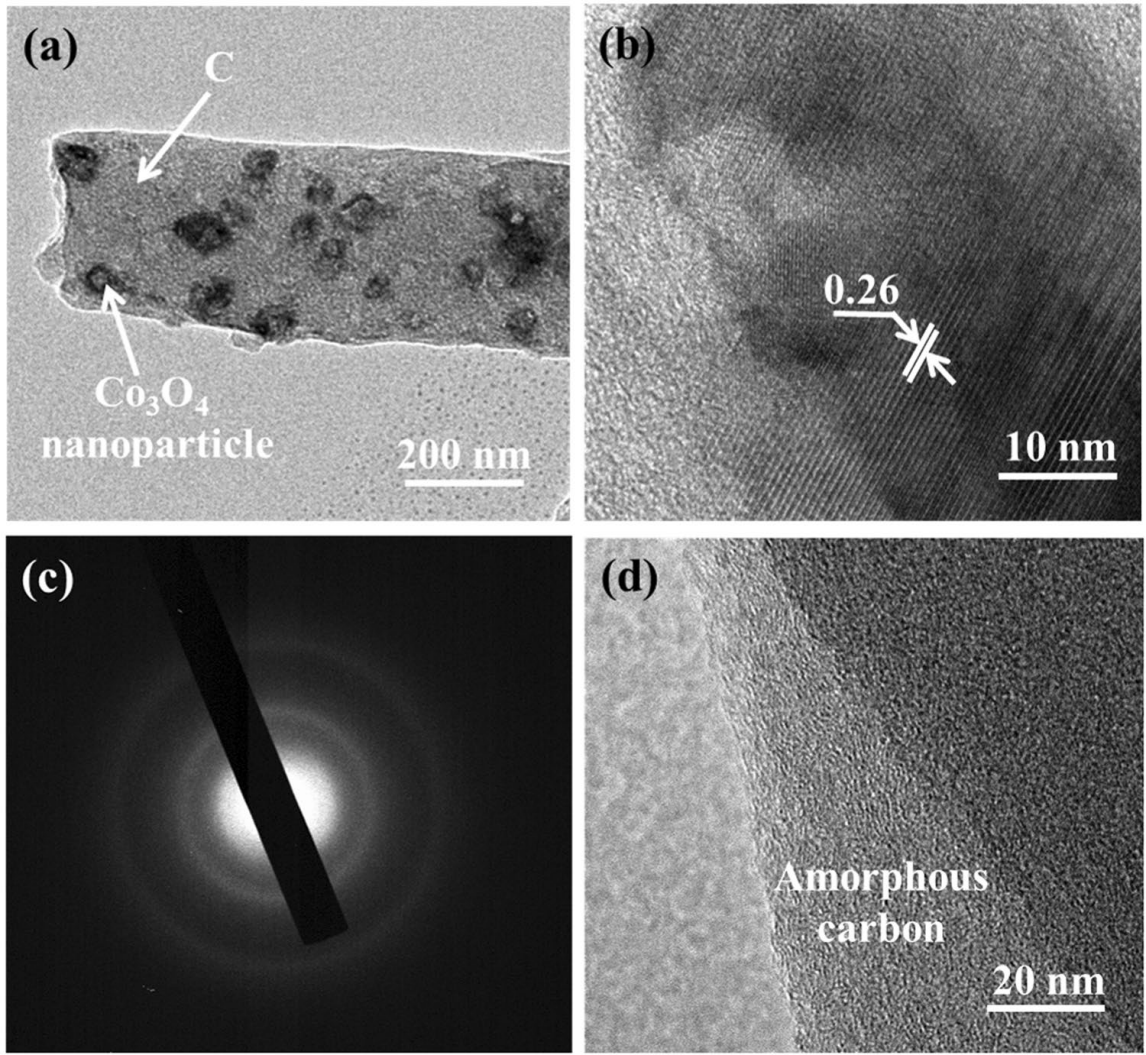
(**a**) HR-TEM images of CA4-800, (**b**) showing lattice distance of CA4-800, (**c**) SAED pattern and (**d**) the image shows amorphous carbon nanofiber layer.

To confirm the presence of cobalt oxide nanoparticles and their uniform distribution in a carbon matrix, TEM analysis with selected area electron diffraction (SAED) was conducted. Figure 3 exhibits the TEM images of CA4-800 composite membrane after calcination at 800 °C. Figure 3a represents the complete structure of individual carbon nanofiber and there is also a uniform dispersion of cobalt oxide nanoparticles in the carbon nanofibers matrix. CNFs/Co_3_O_4_ composites nanofibers are definitely detectable by HR-TEM instrument, displaying a lattice distance of 0.26 nm which is presented in Fig. 3b. The mysterious lattice edge of the nano-shell and clear diffraction circle shape is demonstrated in Fig. 3c, indicating the presence of ordered carbon layers and Co_3_O_4_ nanoparticles. The carbon matrix required areas with different levels of carbon layers is also shown in Fig. 3d.

**Figure 4:**
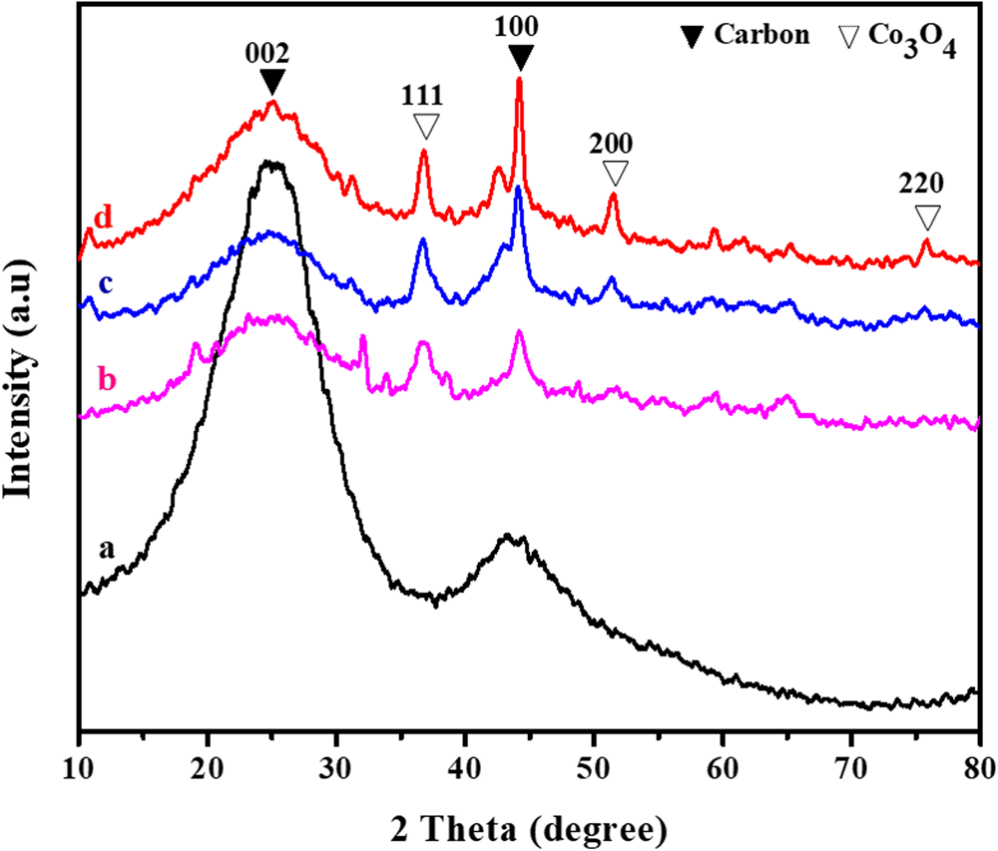
Representative XRD graphs of (**a**) CNFs-800, (**b**) CA2-800, (**c**) CA3-800 and (**d**) CA4-800 samples.

The phase composition of the samples was confirmed by XRD examination. Figure 4 illustrates the patterns of pure CNFs-800, CA2-800, CA3-800, and CA4-800 samples. As estimated, it can be noted that the positions and relative intensities of the diffraction peaks are well corresponded with carbon and standard Co_3_O_4_ patterns, representing a successful synthesis of Co_3_O_4_ and carbon nanofiber composite^23^. The broad diffraction peak at a 2θ of 44° is assigned to the specific reflection (100) diffraction planes of ordered graphitic carbon. An additional broad diffraction peak near to 25° is specified to the characteristic (002) reflection of amorphous carbon. This diffraction peak proves that the Co_3_O_4_ nanoparticles are decomposed and carbothermally reduced to carbon nanofiber to Co_3_O_4_. After the carbonization process in N_2_ flow, three peaks at 2θ angles of approximately 36.12°, 52° and 76° are observed, which can be corresponded to the (111), (200) and (220) reflections of the cobalt metal oxide nanoparticles. In addition, the size of Co_3_O_4_ nanoparticles is calculated to be about 110 nm.

**Figure 5:**
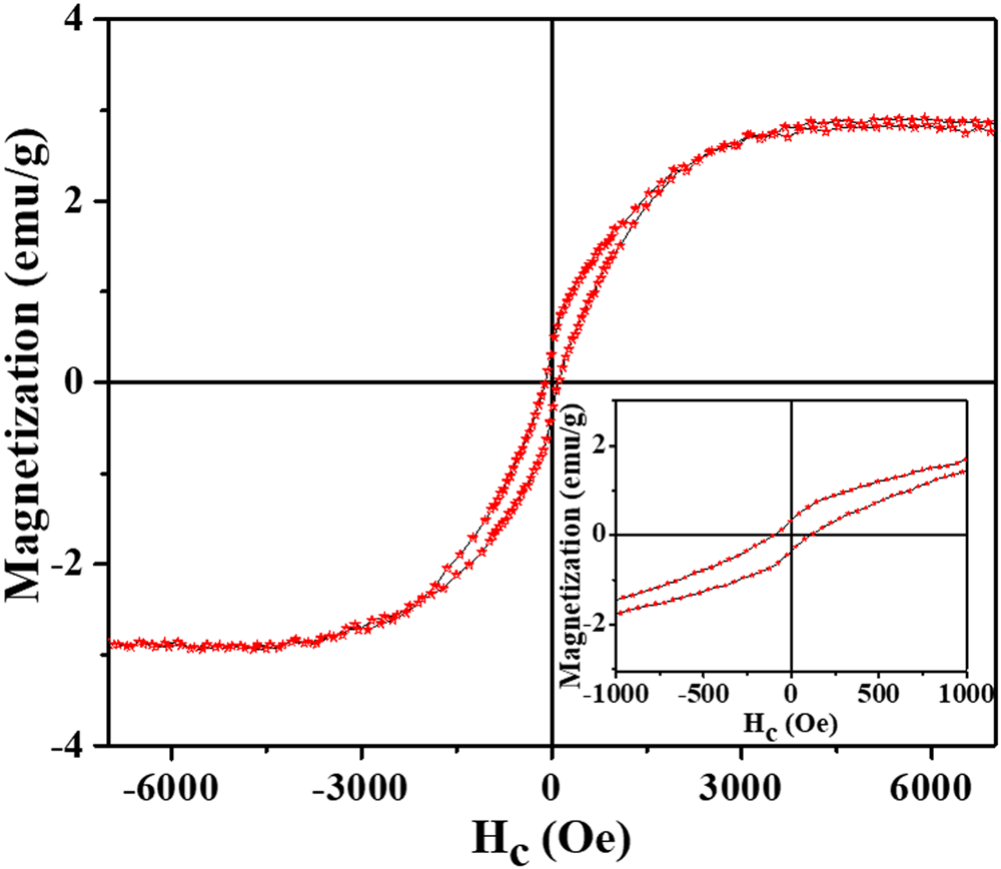
Magnetization curve of CA4-800 sample measured at room temperature. Inset is the magnified image of the considered magnetic property.

The magnetic property of the sample was tested by vibrating sample magnetometer (VSM) instrument. The comprehensible magnetic properties of CA4-800 membrane including saturation magnetization (*M*_*s*_) and coercivities (*H*_*c*_) were measured at room temperature, which has been presented in Fig. 5. As can be realized, the sample exhibits representatively a soft ferromagnetic material behavior. The room temperature measured *M*_*s*_ value for as-synthesis composite of CA4-800 is significantly higher compared with G-Co_3_O_4_ nanocomposites in same condition^24^. Furthermore, the *M*_*s*_ of nanofiber may also decrease due to the larger specific area^25^, and increasing the particle size^26^ result in an increase of the nonmagnetic AAc component in nanofibers and high relative complex permeability of CA4-800 composite sample^6,27^. It is noticeable that the *H*_*c*_ and remanence (*M*_*r*_) are concerned and they both have been observed to increase with an increase of nonmagnetic AAc component in nanofibers. It is worth noting that in *M-H* loops the *H*_*c*_ of synthesized composite nanofibers is more improved compared to the corresponding bulk Co metal, and it was observed at 21 nm nanoparticle size as around 104 Oe as clearly shows in the inset graph in Fig. 5. The magnetic properties differences which are dependent on particle size could be attributable to the surface rotations of the small cobalt oxide nanoparticles. Furthermore, Fig. S3 shows the statistic histogram of Co_3_O_4_ nanoparticle size which is about 21 nm. The size of particles largely influences the magnetic properties of the EM wave absorber. The magnetization increases obviously for nanoparticle sizes approximately less than 10 nm^26^. This particle size will be a very active parameter for EM absorbing and also the percentage of nanoparticle in the composite content. Generally, magnetic nano-materials have a larger *H*_*c*_ than corresponding bulk materials as a result of the significant rise of the surface anisotropy field influenced by the small size effect^5,28^. Besides, the CA4-800 membrane could be facilely manipulated by small magnetic (Fig. S4 and Movie S2).

The nanofiber membranes containing paraffin were used to fabricate EM wave absorber materials. The absorption performance of the composites can be completely attributed to nanofibers’ contribution due to its low permittivity and permeability of paraffin in the composite. The chosen nanofiber samples are CNFs-800 and CAc-800 (c = 0, 2, 3 and 4 wt.% to analyze the effect of AAc content and the effect of nanofibers/paraffin ratio). A key factor for the sample preparation is whether nanofibers can be uniformly dispersed in the paraffin. In this research, nanofibers were loaded with AAc nanoparticles, which are promising to reduce the entanglement among the fibers.

**Figure 6:**
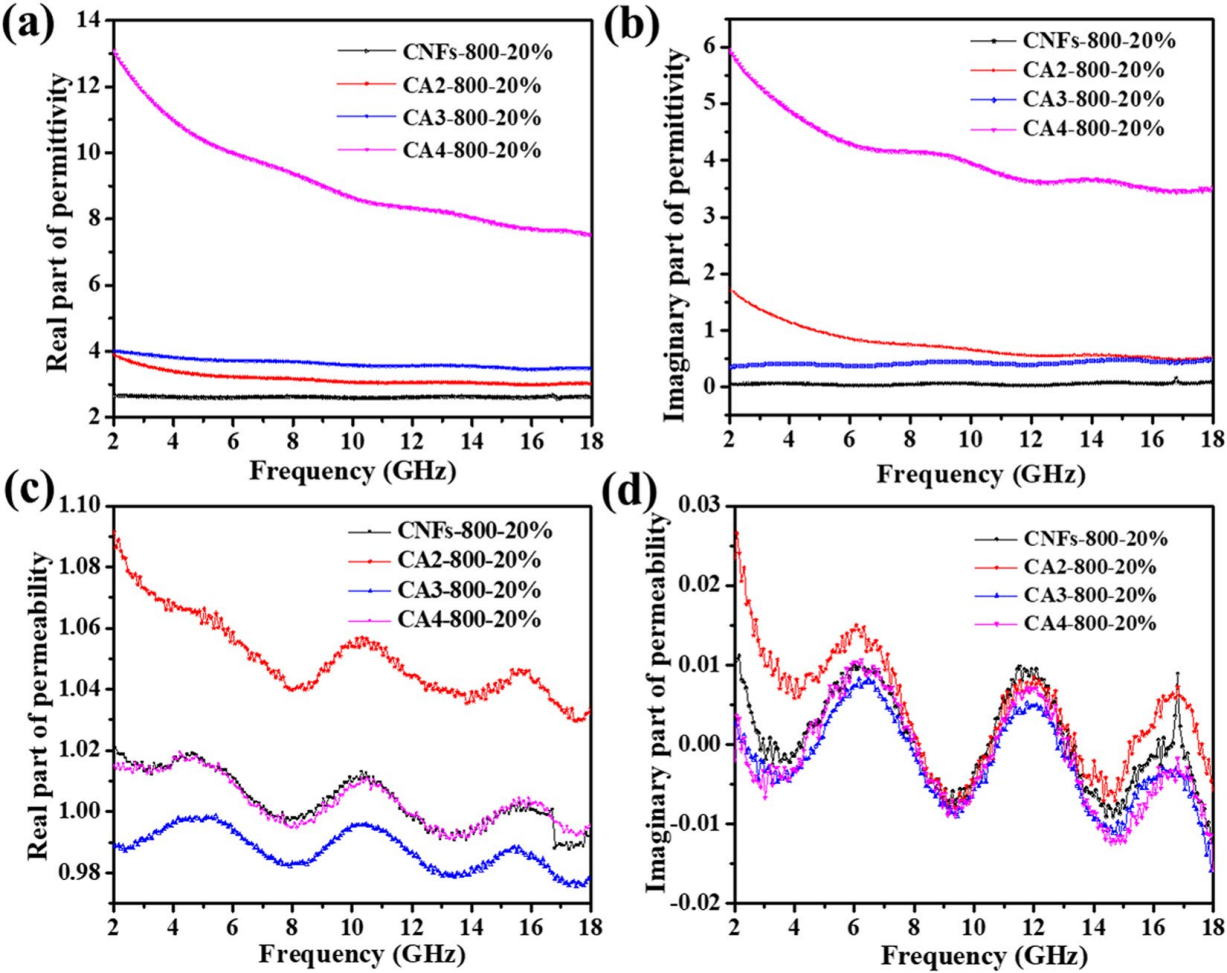
Frequency dependence of (**a**) the real part, (**b**) imaginary part of the relative complex permittivity, (**c**) the real part and (**d**) imaginary part of relative complex permeability of the absorbers in the frequency range 2–18 GHz.

EM parameters for absorbers, including real and imaginary parts of permittivity (ε′, ε″) and permeability (μ′, μ″), are of very importance to evaluate the EM wave absorption properties and represent the ability in accordance to waste EM wave energy^6,29^. Figure 6 shows the ε′ and ε″ of all CNFs-800, CA2-800, CA3-800, and CA4-800 composites in the frequency range 2−18 GHz, which indicates that the CA4-800-20 sample exhibited the highest permittivity among all of them. It can be seen that μ′ and μ″ are less than 1 and change slightly with frequency, thus the wasting of EM wave energy is attributing to its dielectric loss. For comparisons, the ε′, ε″ of CNFs-800-20 sample and CAc-800 sample (c = 3 and 4) are depicted in Fig. 6(a–d) respectively. It is apparent that both real part ε′ and imaginary part ε″ demonstrate a spectrum characteristic and their values decrease as frequency increases. The ε′, ε″ and μ′, μ″ for pure carbon fibers are relatively lower as compared to CAc-800-20 sample. For samples with different AAc contents increase from 0 wt.% to 4 wt.%, both ε′ and ε″ increase correspondingly as well, as shown in Fig. 6(a).

**Figure 7:**
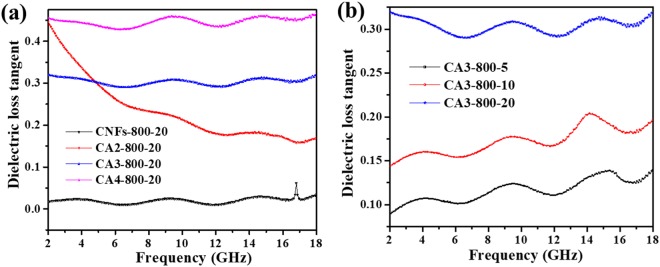
Dielectric loss tangent of (**a**) CNFs-800-20, CA2-800-20, CA3-800-20 and CA4-800-20 (**b**) CA3-800-p (p = 5, 10 or 20) in the frequency range 2-18 GHz.

The ratio of fiber/paraffin is also vital to the EM performance. Especially, ε′ and ε″ of CNFs-800-20 are closer to each other than any other samples, implying a higher dielectric loss, which is beneficial for enhancing the EM wave absorption. The ε′ and ε″ of CNFs-800, CA2-800 and CA3-800 remain almost constant along the entire frequency range except for CA4-800 sample. ε″ values are very close to zero, representing a poor dielectric loss of sample (Fig. 7). With increasing the AAc amount in composite samples, both ε′ and ε″ are obviously enhanced and become more and more dependent on the frequency. For example, the ε′ values of CNFs-800-20, CA2-800-20, CA3-800-20, and CA4-800-20 decrease from 2.7 to 2.6, 3.8 to 3.0, 4.0 to 3.5 and 13.0 to 7.54, respectively; the ε″ values of CNFs-800-20, CA2-800-20, CA3-800-20, and CA4-800-20 decline from 0.01 to 0.08, 1.7 to 0.5, 0.4 to 0.47, and 6.0 to 3.5, respectively. ε″ of CNFs-800 has a variation because it is pure carbon nanofibers with no AAc content. All these results demonstrate that AAc plays a dominant role in determining the dielectric loss properties of these materials.

Dielectric loss tangent (tan δ_ε_) and magnetic loss tangent (tan δ_μ_) can be used to measure and characterize the EM energy loss in the absorber material. The EM wave improves when the tan δ_ε_ of the material increases, due to a higher attenuation element. In this work, CA4-800-20 samples present a greater absorption properties as compared with other samples. Moreover, the reflection loss can be influenced by several factors, and the absorption mechanisms are discussed as follows. The EM wave absorption of the composite samples depends on their dielectric loss, thus the tan δ_ε_ and tan δ_μ_ values were calculated and discussed for all samples and the results are given in Figs 7 and S5 respectively.

For the CNFs-800-20 absorber material demonstrated in Fig. 7(a), the tan δ_ε_ of CNFs-800-20 is close to 0.45 at the most frequency range between 2–18 GHz. For samples with AAc addition, the values of tan δ_ε_ are around 0.3–0.45 for CAc-800-20 (c = 2, 3 or 4) in the full range of 2–18 GHz. Figure 7a demonstrates the dielectric loss tangent of composite samples as a well-designed of frequency. Figure 7b displays the dielectric loss tangent increase from about 0.09 to 0.3 as CA3-800-p (p = 5, 10 or 20). When samples were mixed with 20 wt.% paraffin, the loss tangent are much increased and also show frequency dependence on the measured frequency range, especially for the high-frequency area from 10 to 18 GHz. As a consequence, it is noticeable that the composite nanofibers’ dielectric loss is obviously more upraised than pure carbon nanofibers material. The enhanced EM wave absorbing performance can be realized as a result of introducing a suitable amount of AAc. Furthermore, though tan δ_ε_ for CA4-800-20 is slightly less than that for CA3-800-20, they show lower R_L_ values than CA3-800-20, indicating a better impedance match with free space impedance. The EM wave absorption materials are not always excellent for higher permittivity and permeability. It is the most important matter to meet the requirement of matching material impedance with free space^30^. Due to the complex effect of Co_3_O_4_ nanoparticles, impedance matching condition is greatly enhanced and thus the absorber samples exhibit improved absorption performance^31^.

By the study of composite absorbers principle in EM wave field, the R_L_ was calculated using subsequent equations^2,6,32–34^ and Matlab software.

1 $${R}_{L}{\rm{dB}}=20\,\mathrm{log}|\frac{{{\rm{{\rm Z}}}}_{in}-{{\rm{{\rm Z}}}}_{0}}{{{\rm{{\rm Z}}}}_{in}+{{\rm{{\rm Z}}}}_{0}}|$$

2 $${{\rm{{\rm Z}}}}_{in}=\sqrt{\frac{{\mu }_{r}}{{\varepsilon }_{r}}}\,\tan \,h(j\,\frac{2\pi fd}{c})\sqrt{{\mu }_{r}{\varepsilon }_{r}}$$

3 $${\varepsilon }_{r}={\rm{\varepsilon }}^{\prime} -j{\rm{\varepsilon }}^{\prime\prime} $$

4 $${\mu }_{r}=\mu ^{\prime} -j\mu ^{\prime\prime} $$

where *Z*_*in*_, *Z*_0_, c, *f* and d are the input impedance at the material and air interface, the source impedance, the EM wave velocity in free space, the frequency of the incident wave, the thickness of the absorbing material. ε′ is the real part of the permittivity, ε″ is the imaginary part of the permittivity, μ′ is the real part of permeability, μ″ is the imaginary part of permeability. Therefore, *ε*_*r*_ and *μ*_*r*_ are relative complex permittivity and permeability.

**Figure 8:**
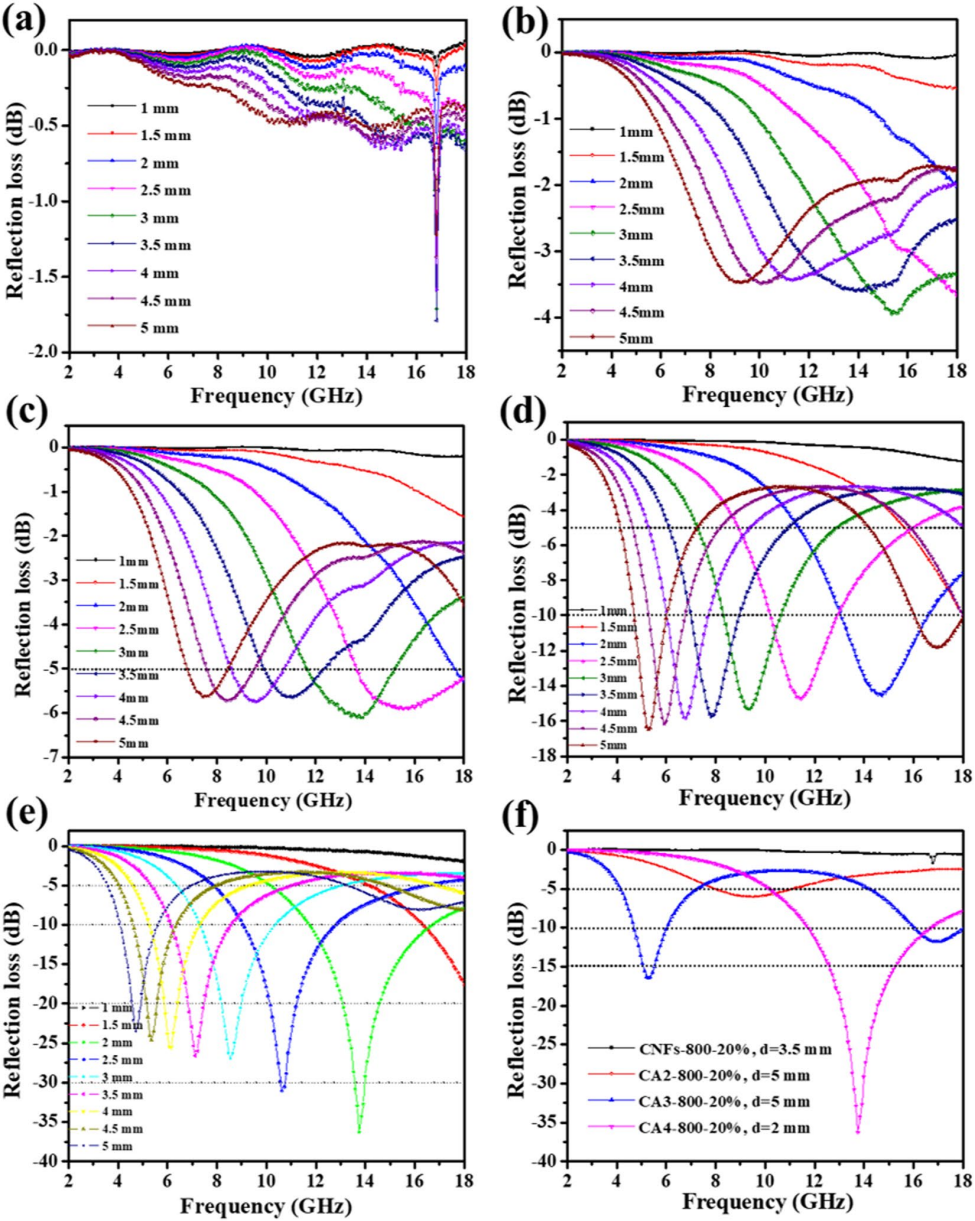
Reflection loss of (**a**) CNFs-800-20, (**b**) CA3-800-5, (**c**) CA3-800-10, (**d**) CA3-800-20, (**e**) CA4-800-20 with different thickness (1-5mm) in frequency range of 2-18 GHz and **(f**) CNFs-800-20 (d = 3.5 mm), CA2-800-20 (d = 5 mm), CA3-800-20 (d = 5 mm), CA4-800-20 (d = 2 mm).

The absorption wave is incidence on the absorber sample backed perfect conductor, the relation between the matching thickness (*d*) and frequency (*f* ) is obviously expressed by the equation.: $f=\frac{nc}{4d}\sqrt{|\varepsilon ||\mu |}$ (n = 1, 3, 5…). Figure 8 displays the frequency dependence of reflection loss of different thickness Co_3_O_4_/carbon composite nanofibers. Detailed experimental results are shown in Fig. 8a–f, demonstrating the R_L_ during of 2–18 GHz range at given thickness of 1–5 mm. The R_L_ of equal and less than −20 dB are accomplished along the 4.5–14.4 GHz ranges. Moreover, the R_L_ values of absorber materials depend on their thicknesses, suggesting that the absorbability of the sample can be successfully and simply changed by controlling the thickness. The absorption bandwidths with R_L_ less than −5 dB are from 6.3 to 18 GHz for CA3-800-10, from 4 to 18 GHz for CA3-800-20 and from 3.2 to 18 GHz for CA4-800-20 during of 1–5 mm thicknesses.

It is worth noting that CA4-800-20 possess a wide frequency range and a thin absorber matching layer thickness (R_L_ <−20 dB). As obtainable in many related work in nano-composites summarized in Table S1, the EM wave absorbing performances of presented CA4-800-20 sample is compared with the CoNi/C nano-capsules^18^, porous C/Co^35^, MWCNTs/Co^36^, FeCo/CNTs^37^, FeCo@C^19^ and Co/C-tubes^38^ magnetic samples. The results demonstrate that the lightweight CA4-800-20 sample exhibit outstanding wave absorbing properties into a wide range of C-band to Ku-band matching with a lower mass density. Furthermore, the minimum R_L_ values of 16.48 and 36.27 dB are obtained at 5.28 and 13.76 GHz and 5 and 2 mm for CA3-800-20 and CA4-800-20, respectively. This minimum R_L_ value of CA4-800-20 may be ascribed to the excellent magnetic properties. The pure carbon nanofibers under the same operating conditions were investigated so as to compare with the composite sample, which is demonstrated in Fig. 8a.

The CNFs-800 membrane is displays −1.8 dB at ranging (2–18) GHz demonstrating a lower EM absorbing value than that of CA3-800-20 and CA4-800-20 composite samples because of the absence of magnetic content in the composite. Thus, it is worth noting that the absorber’ thickness is one of the most important parameters which largely affects the frequency at the minimum reflection loss^39^. Table S2 shows the microwave absorption of samples with different AAc contents and different thicknesses. The advanced absorbing performance of composites is mostly due to a good EM impedance matching because of their relatively low permittivity. These results fully indicate that the as-prepared CA3-800 and CA4-800 composite nanofibers are capable of serving as exceedingly effective absorbers with lower thicknesses. Consequently, the high absorbed EM wave into the composite sample is due to its lower reflected wave on the surface. We synthesized and characterized microwave absorption composite membranes in a wide frequency range and different thicknesses, which exhibited different capabilities to increase the absorbance of EM wave. Figure S6 shows the schematic diagram of EM wave reduction through nanofibers as a shield. When the R_L_ values of the sample are below −10 and −20 dB, they correspond to the bigger percentage of the wave (almost 90 and 99%, respectively) that are absorbed. This R_L_ value is considered as a representative objective for effective absorbance from a view of practical application^40–42^.

Moreover, the as-prepared CA2-800-5, CA2-800-10 and CA2-800-20 samples in different thicknesses mainly contributed to a low EM reflection loss in entire frequency range due to their lower AAc content. It is worth noting that CA2-800-20 possess a wide frequency range (R_L_<−5 dB) compared with the same sample with a lower paraffin content. Additionally, the minimum R_L_ values of 1.90, 3.08 and 6.07 dB are obtained at 17.12, 10.32 and 9.52 GHz with a matching thickness of 3.5, 5 and 5 mm for CA2-800-5, CA2-800-10 and CA2-800-20, respectively. The sample with less AAc content demonstrates the lower reflection loss values (Fig. S7). SEM images and statistic histogram of fiber length distributions for CA3-800, CA4-800 composite membranes are compared in Fig. S8. For microwave test, the fiber length of these two membranes were 1.5 and 1.24 μm, respectively. A shorter fiber length can absorb more amount of wave and thus contribute to a better electromagnetic wave absorbing performance.

## Conclusion

In this work, Co_3_O_4_/carbon composite nanofibrous membranes have been successfully constructed using combined techniques of electrospinning, stabilization and carbonization processes. Electromagnetic (EM) absorbing behavior of the nanofibrous membranes was studied as EM wave absorbers in the frequency range of 2–18 GHz. The obtained samples exhibit improved absorbing performance towards higher frequencies with an increase in the cobalt nanoparticle concentration. Carbon nanofibers have reduced the magnetization and coercivity of cobalt nanoparticles which can finally cause a decrease in magnetic permeability in composite membranes. Co_3_O_4_/carbon nanofibrous material mixed with 20 wt.% of paraffin affords superior absorption performance compared with pure carbon nanofibrous with the same content of paraffin. The optimal R_L_ is 36.27 dB with a coating thickness of 2 mm in 13.76 GHz. The R_L_ of the composites is considered as a result of the dielectric loss of EM wave field in the composites samples. All above results indicated that the resultant Co_3_O_4_/carbon composite membranes exhibit excellent EM properties and such composite nanofibers could be served as attractive EM wave absorbing materials. Additionally, the fabricated materials are environmental friendly, approachable and low-cost. The justified concept of nanofibrous membrane enabled microwave absorbing materials can be extended to other common practical applications in magnetic and electromagnetic shielding system, which is promising and could possibly change the way of current EM wave absorption technology

## Experimental

### Materials usage

Polyacrylonitrile (PAN, Mw = 150 000) polymer was supplied by Shanghai Aladdin Chemical Co., China. Dimethylformamide (DMF) solvent and cobalt acetylacetonate (AAc) (Co(acac)_3_) powder were produced from Shanghai Chemical Reagents Co., Ltd., China.

### Fabrication of PAN/AAc membranes

In a typical procedure, the fabrication of precursor fiber was synthesized by co-precipitation method^23^. 10 wt.% of PAN polymers were mixed with DMF solvent to obtain PAN/DMF solutions with an AAc content of 2 wt.% (AAc/PAN ≈ 2/10), 3 wt.% (AAc/PAN ≈ 3/10) and 4 wt.% (AAc/PAN ≈ 4/10) respectively. After 10 hrs vigorous magnetic stirring at room temperature, uniform reddish sticky solutions (AAc/PAN) with AAc content of 0, 2 wt.%, 3 wt.% and 4 wt.% were successfully prepared. The electrospinning procedure was performed with an applied voltage of 25 kV and solution flow rate of 1mL h^−1^. The distance between the needle tip and metallic roller covered with nonwoven was kept at 20 cm. The temperature and humidity of the electrospinning compartment were maintained at 25 ± 2 °C and 45 ± 5%, respectively. Pure PAN nanofibers were manufactured by the similar way for comparison.

### Fabrication of the composite samples

The nanofibrous membranes were dried at 80 °C for 1 hr using a vacuum to eliminate the solvent, and then the sample was stabilized for 2 hrs from room temperature to 280 °C at a rate of 5 °C/min. After that, the obtained stabilized nanofibrous samples were annealed at a heating rate of 2–3 °C min^−1^ to a specified temperature of 800 °C using a tube furnace with the N_2_ flow and kept for 2 hrs to complete the carbonization process.

### Preparing of the absorber materials

The EM wave absorber materials were finally prepared by mixing the composite nanofibers with paraffin. Pure carbon nanofibers of CNFs-800 along with composite cobalt/carbon nanofibers of CAc-800 (c refers to AAc content and equals to 2, 3 or 4) were selected to analyze the EM wave absorption properties. CNFs-800 nanofibers were added into paraffin in 20 wt.% to obtain the testing sample of CNFs-800-p (p refers to nanofiber/paraffin ratio and equals to 20). The EM absorption testing samples of CAc-K-p was also correspondingly prepared, when K = 800, p = 5, 10 or 20 and c = 2, 3 or 4.

### Characterization and measurements

The morphology of as-synthesized samples was studied using FE-SEM (S-4800, Hitachi Ltd., Japan) successively operating at 2 kV and 5 kV. High-resolution transmission electron microscopy (HR-TEM, JEM-2100F, JEOL Ltd., Japan) was chosen to characterize the fibers’ nanostructures, including selected area electron diffraction (SAED). The phase structures of the samples were characterized using X-ray diffraction (XRD) (TD-3500 Dandong Tongda Science and Technology Co., China. Cu Kα, λ = 1.5406 Å). The magnetic properties of the sample were confirmed with a vibrating sample magnetometer instrument at room temperature.

### EM absorption measurement

The CNFs/Co_3_O_4_ composite samples used for EM wave absorption measurements were prepared by mixing the resultant samples with different mass percentages of paraffin wax. The mixtures were then pressed into a cylindrical-shaped sample (Φ_out_ = 7.00 mm and Φ_in_ = 3.04 mm). The permittivity and permeability values were realized in the frequency of 2–18 GHz with a coaxial wire method by an Agilent PNA-N5244A vector network analyzer^33^. Reflection loss (R_L_) was finally calculated by the Matlab software in accordance with the resultant data to evaluate the EM absorption performance.

## Electronic supplementary material

Supplementary Information

Supporting Movie S1

Supporting Movie S2
