# Supplementary material for: Co3O4/carbon composite nanofibrous membrane enabled high-efficiency electromagnetic wave absorption
Source: Sci Rep. 2018 Aug 17;8:12402. doi: 10.1038/s41598-018-30871-2 (PMC6097987; doi:10.1038/s41598-018-30871-2)
Supplement: Supplementary file 1 — Supplementary Information [file 41598_2018_30871_MOESM1_ESM.docx]

**SUPPORTING INFORMATION**

**Co_3_O_4_/carbon composite nanofibrous membrane enabled high-efficiency electromagnetic wave absorption**

Ibrahim Abdalla^1^, Jiali Shen^2^, Jianyong Yu^3^, Zhaoling Li^2,3,^*, Bin Ding^1,2,3,^*

State Key Laboratory for Modiﬁcation of Chemical

Fibers and Polymer Materials

College of Textiles

Donghua University

Shanghai 201620, China

E-mail: binding@dhu.edu.cn

Prof. J. Yu, Prof. B. Ding

Nanoﬁbers Research Center

Modern Textile Institute

Donghua University

Shanghai 200051, Chin

State Key Laboratory for Modiﬁcation of Chemical

Fibers and Polymer Materials

College of Textiles

Donghua University

Shanghai 201620, China

E-mail: binding@dhu.edu.cn

Prof. J. Yu, Prof. B. Ding

Nanoﬁbers Research Center

Modern Textile Institute

Donghua University

Shanghai 200051, Chin

State Key Laboratory for Modiﬁcation of Chemical

Fibers and Polymer Materials

College of Textiles

Donghua University

Shanghai 201620, China

E-mail: binding@dhu.edu.cn

Prof. J. Yu, Prof. B. Ding

Nanoﬁbers Research Center

Modern Textile Institute

Donghua University

Shanghai 200051, Chin

^1^ State Key Laboratory for Modification of Chemical Fibers and Polymer Materials, College of Materials Science and Engineering, Donghua University, Shanghai 201620, China

^2^ Key Laboratory of Textile Science and Technology, Ministry of Education, College of Textiles, Donghua University, Shanghai 201620, China

^3^ Innovation Center for Textile Science and Technology, Donghua University, Shanghai 200051, China.

*Correspondence to: zli@dhu.edu.cn (Prof. Z. Li), binding@dhu.edu.cn (Prof. B. Ding)

**
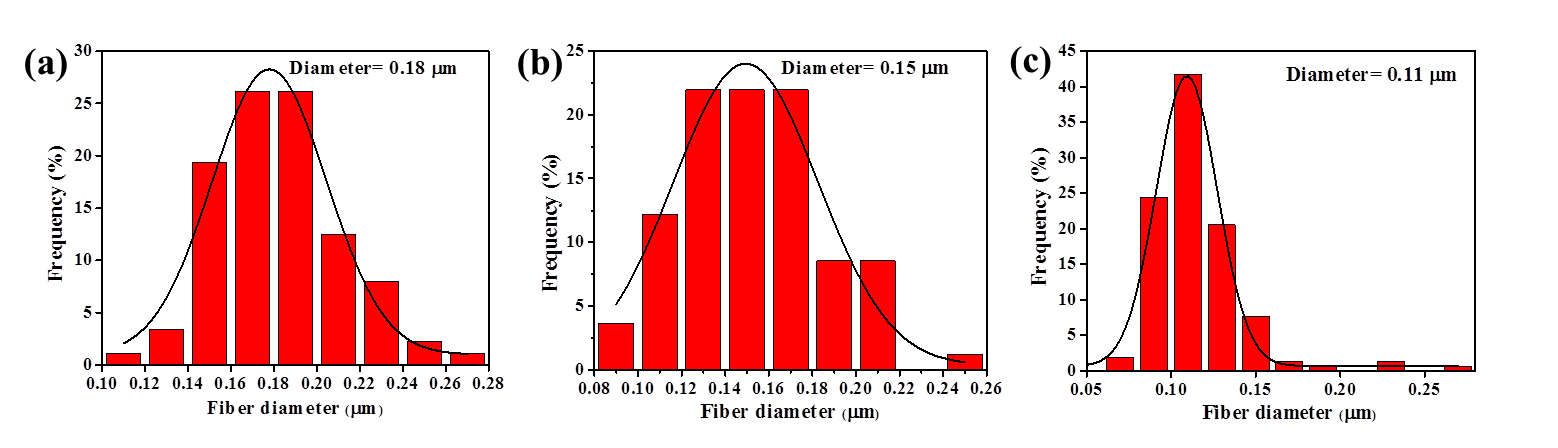
**Fig. S1 Statistic histogram of fiber diameters of (a) CA2-800, (b) CA3-800, (c) CA4-800 samples.


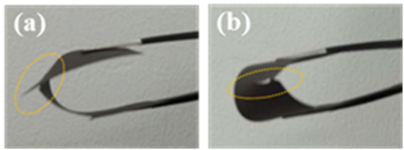


Fig. S2 The digital images indicating the flexibility of (a) the pure sample (CNFs-800) and (b) composite sample with a lower concentration of nanoparticles (CA2-800).





Fig. S3 The statistic histogram of Co_3_O_4_ nanoparticle size.


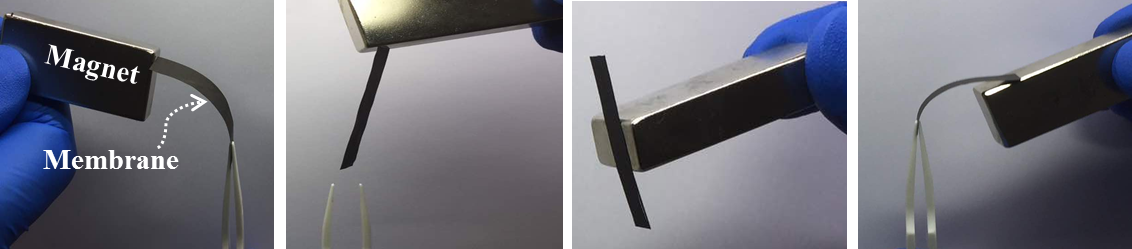


Fig. S4 Photographs indicating the CA4-800 membrane could be facilely manipulated by a small magnetic.


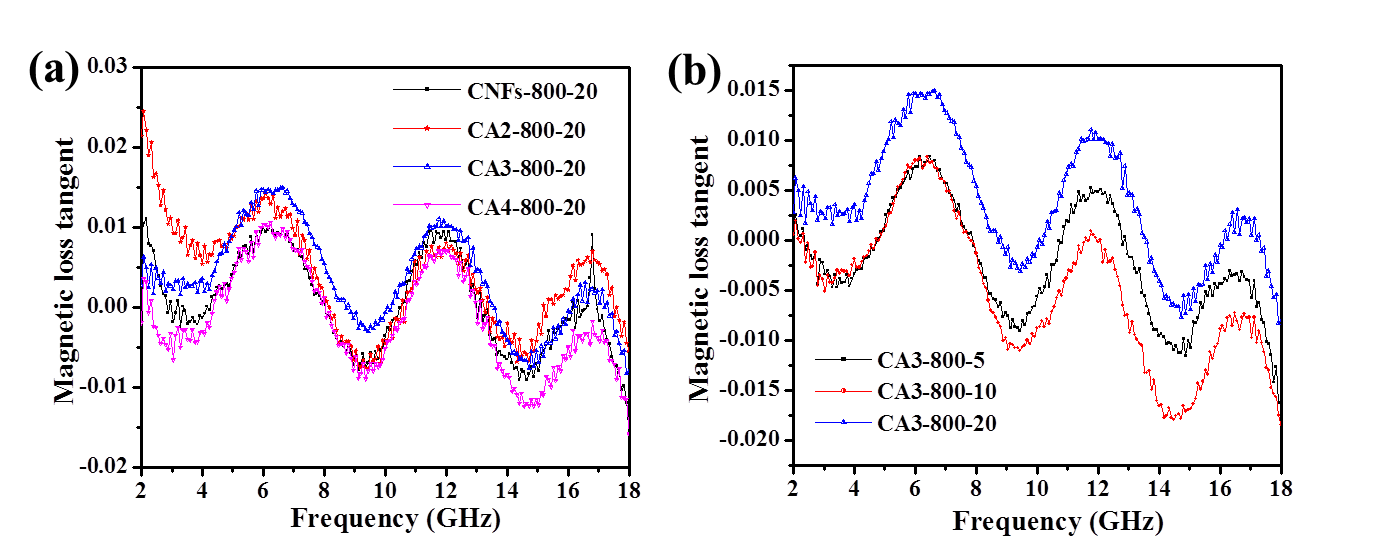


Fig. S5 Magnetic loss tangent of (a) CNFs-800-20, CA2-800-20, CA3-800-20, CA4-800-20and (b) CA3-800-p (p=5, 10 or 20) in the frequency range 2-18 GHz.

**
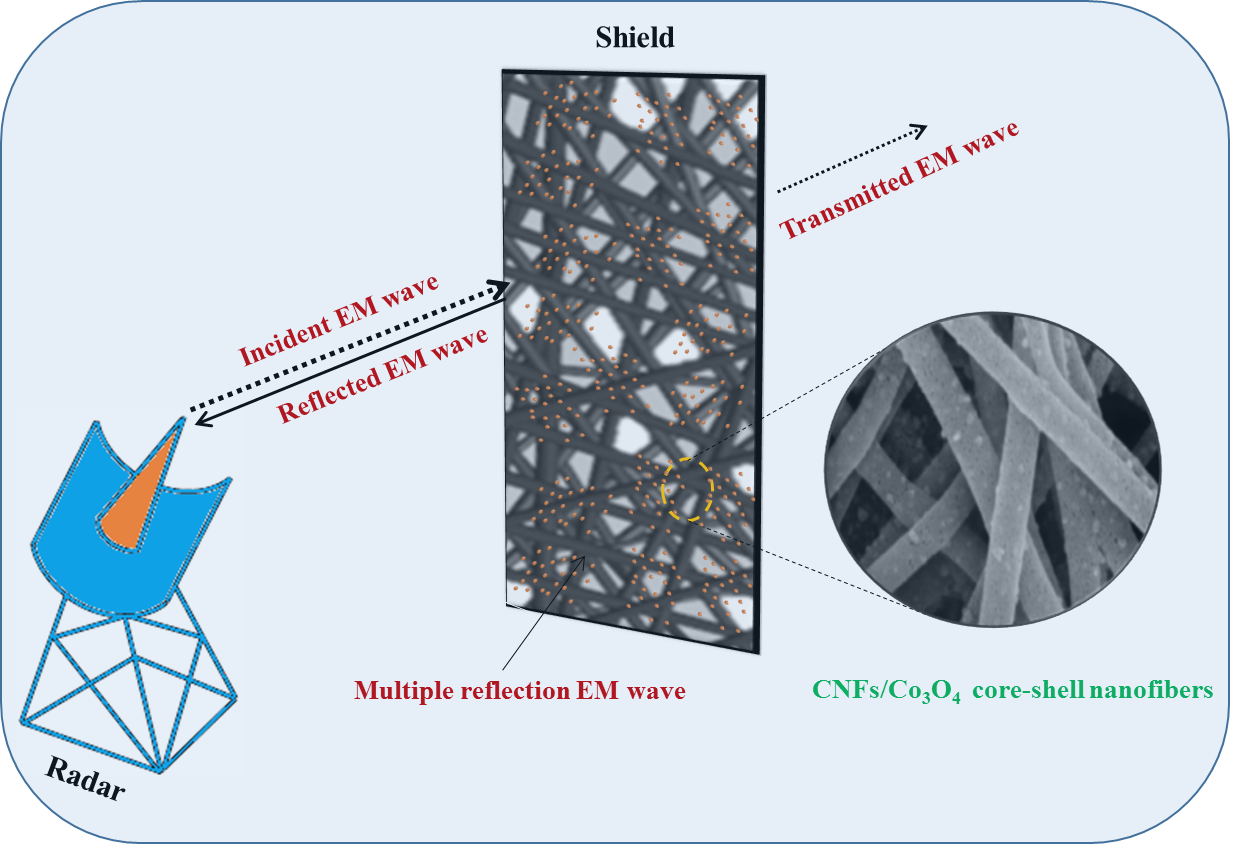
**

Fig. S6 Schematic diagram of EM wave reduction through nanofibers as a shield.

**
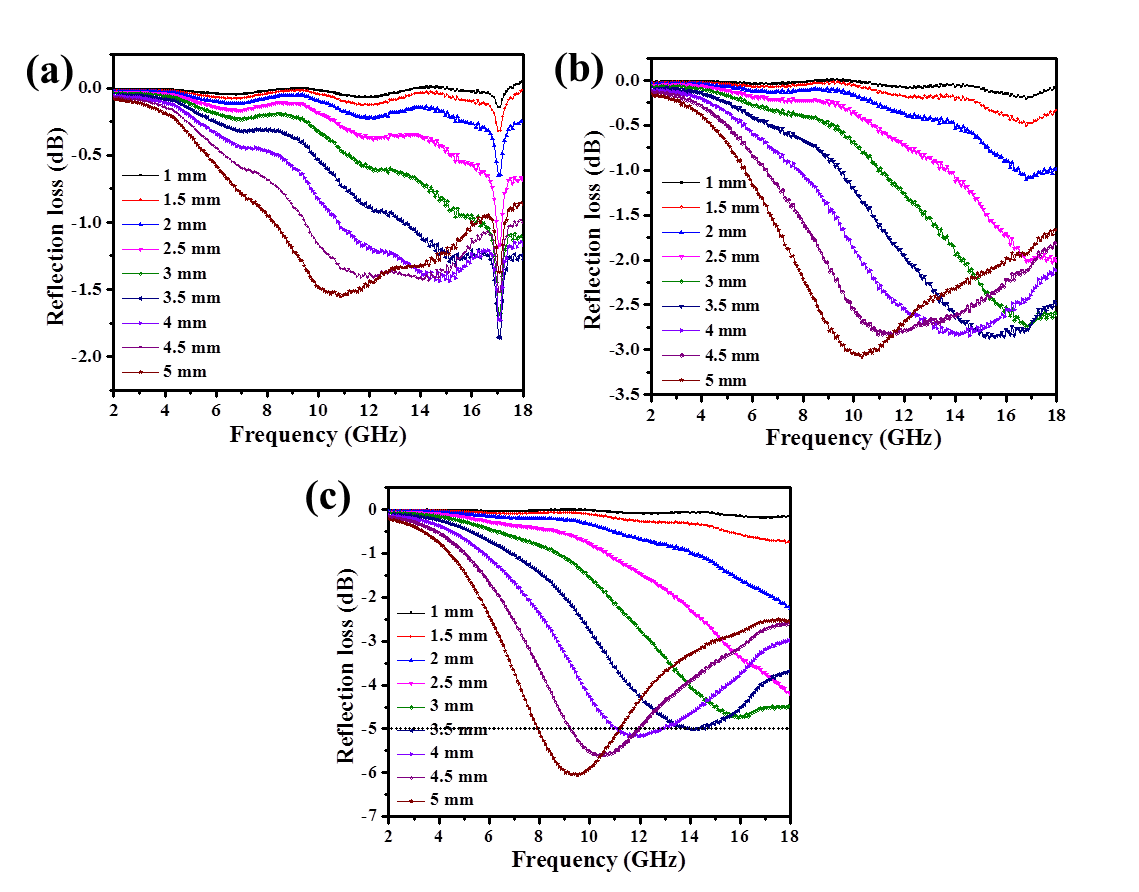
**

Fig. S7 Reflection loss of (a) CA2-800-5, (b) CA2-800-10, (c) CA2-800-20 with different thickness (1-5mm) in frequency range of 2-18 GHz


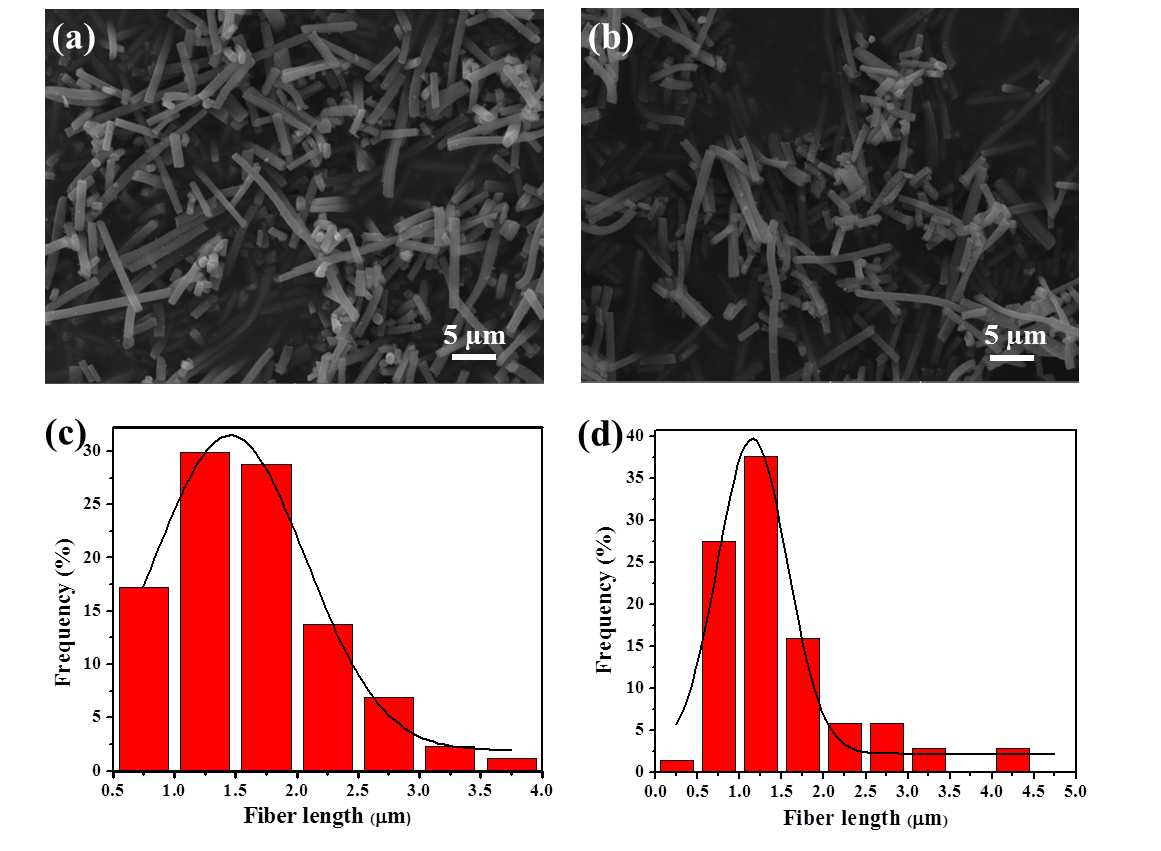


Fig. S8 SEM images of (a) CA3-800, (b) CA4-800 and statistic histogram of fiber length distribution of (c) CA3-800 and (d) CA4-800 samples.

| **Sample** | **Mass Ratio**  **(wt.%)** | **Frequency range**  **(R_L_<-20 dB)**  **(GHz)** | **Minimum R_L_** | | **References** |
| --- | --- | --- | --- | --- | --- |
|  |  |  | **Value (dB)** | **Matching**  **Thickness (mm)** |  |
| CoNi/C nanocapsules | 40 | 5–17 | -35 | 2.0 | ^18^ |
| Porous C/Co | 30 | 4–10 | -40 | 5.0 | ^35^ |
| MWCNTs/Co | 60 | 2.35–3.51 | -37 | 5.25 | ^36^ |
| FeCo/CNTs | 30 | 9–15.5 | -37.3 | 8.0 | ^37^ |
| FeCo@C | 50 | 2-6 | -29 | 3.8-8.5 | ^19^ |
| Co/C-tubes | 30 | 4-9 | -40 | 2.5-5 | ^38^ |
| CA4-800-20 | 20 | 4.56-14.56 | 36.3 | 2 | This work |

Table S1 EM wave absorption properties of some recently reported researches.

| ***d* (mm)** | **<-5 dB Bandwidth (GHz)** | | | **<-10 dB Bandwidth (GHz)** | | | **Minimum absorption (dB)** | | |
| --- | --- | --- | --- | --- | --- | --- | --- | --- | --- |
|  | **c=2** | **c=3** | **c=4** | **c=2** | **c=3** | **c=4** | **c=2** | **c=3** | **c=4** |
| 1 | - | - | - | - | - | - | -0.15 | -1.26 | -1.93 |
| 1.5 | - | 15.6 | 14.24 | - | 18 | 16.32 | -0.75 | -10 | -17.64 |
| 2 | - | 11.36 | 10 | - | 13.12 | 11.76 | -2.25 | -14.52 | **-36.27** |
| 2.5 | - | 8.88 | 7.92 | - | 10.24 | 9.04 | -4.21 | -14.73 | -31.06 |
| 3 | - | 7.28 | 6.4 | - | 8.32 | 7.28 | -4.75 | -15.32 | -26.96 |
| 3.5 | 13.84 | 6.08 | 5.36 | - | 6.96 | 6.16 | -5.01 | -15.74 | -26.57 |
| 4 | 10.96 | 5.28 | 4.56 | - | 6 | 5.28 | -5.17 | -15.80 | -25.51 |
| 4.5 | 9.28 | 4.64 | 4 | - | 5.28 | 4.64 | -5.62 | -16.17 | -25 |
| 5 | 7.92 | 4.16 | 3.6 | - | 4.72 | 4.08 | -6.05 | -16.48 | -23.53 |

Table S2 A comparison of microwave absorption for CAc-800-20 (c=2, 3, 4) samples with different AAc contents and different thicknesses (1-5mm).

**Supporting movie**

Movie S1 A movie presenting the flexibility of CA4-800 membrane.

Movie S2 A movie presenting the CA4-800 membrane could be facilely manipulated by a small magnetic.
